# Accuracy of theoretical IOL formulas for Panoptix intraocular lens according to axial length

**DOI:** 10.1038/s41598-021-86604-5

**Published:** 2021-04-01

**Authors:** Ayoung Choi, Hyunggoo Kwon, Sohee Jeon

**Affiliations:** Keye Eye Center, 326 Teheran-ro, Gangnam-gu, Seoul, Korea

**Keywords:** Lens diseases, Refractive errors

## Abstract

The accuracy of intraocular lens (IOL) calculations is suboptimal for long or short eyes, which results in a low visual quality after multifocal IOL implantation. The purpose of the present study is to evaluate the accuracy of IOL formulas (Barrett Universal II, SRK/T, Holladay 1, Hoffer Q, and Haigis) for the Acrysof IQ Panoptix TFNT IOL (Alcon Laboratories, Inc, Fort Worth, Texas, United States) implantation based on the axial length (AXL) from a large cohort of 2018 cases and identify the factors that are associated with a high mean absolute error (MAE). The Barrett Universal II showed the lowest MAE in the normal AXL group (0.30 ± 0.23), whereas the Holladay 1 and Hoffer Q showed the lowest MAE in the short AXL group (0.32 ± 0.22 D and 0.32 ± 0.21 D, respectively). The Haigis showed the lowest MAE in the long AXL group (0.24 ± 0.19 D). The Barrett Universal II did not perform well in short AXL eyes with higher astigmatism (*P* = 0.013), wider white-to-white (WTW; *P* < 0.001), and shorter AXL (*P* = 0.016). Study results suggest that the Barrett Universal II performed best for the TFNT IOL in the overall study population, except for the eyes with short AXL, particularly when the eyes had higher astigmatism, wider WTW, and shorter AXL.

## Introduction

Modern intraocular lens (IOL) formulas have significantly improved refractive outcomes after phacoemulsification and IOL implantation^1–3^. The clinical importance of postoperative refractive outcomes is most noted in patients with multifocal IOL (MIOL) implantation. Postoperative refractive outcomes that do not achieve emmetropia result in blurred vision and halos in these patients. Accordingly, many studies have verified the accuracy of the theoretical IOL formula for each MIOL^2,3^.

One of the most commonly mentioned diffractive MIOLs in literature is the Acrysof IQ Panoptix TFNT IOL (Alcon Laboratories, Inc, Fort Worth, Texas, United States); this MIOL has consistently shown favorable clinical outcomes since it has been introduced in the market in 2015^4–8^. Lawless et al.^6^ reported a mean absolute error (MAE) of − 0.01 ± 0.22 D, and 100% of the eyes were within ± 0.50 D of the intended correction. Kohnen et al.^7^ reported that 93% of eyes were within ± 0.50 D of the intended correction when the Haigis formula was used. Recently, Shajari et al.^8^ compared nine modern IOL formulas and found that the Barrett Universal II formula had the lowest maximum absolute prediction error (0.294 D). Although the accuracy of modern IOL formulas has been verified as mentioned previously, most eyes in previous study population had an axial length (AXL) within the normal range. It is well recognized that the accuracy of IOL calculations is suboptimal for atypical eyes^2,9,10^, evidenced by the fact that less than 75% of even the most accurate formulas provide results within ± 0.5 D of the target^9^. There are only limited data about the accuracy of IOL formulas for MIOLs, including the TFNT IOL, in eyes with extreme ocular metrics, such as high myopia and hyperopia. The purpose of the present study is to (1) evaluate the accuracy of IOL formulas for TFNT IOL implantation based on the AXL from a relatively large cohort of 2018 cases and (2) identify the factors that are associated with inaccurate outcomes.

## Results

Among 2679 eyes of Panoptix IOL implantation, 2018 eyes satisfied the inclusion criteria and were reviewed in the present study. Table 1 shows the baseline characteristics of enrolled patients. Among 2018 eyes, 260 eyes (12.9%) with AXL ≤ 22.5 mm were classified in the short AXL group, 98 eyes (4.9%) with AXL ≥ 26.0 mm were classified in the long AXL group, and 1660 eyes (82.2%) with AXL between 22.5 and 26.0 mm were classified in the normal AXL group. There were significant differences in age, UDCA, and CDVA (*P* < 0.001, *P* < 0.001, and *P* = 0.008, respectively), whereas sex, UNVA, and CNVA showed no difference between groups (*P* = 0.867, *P* = 0.113, and *P* = 0.116, respectively). The anterior chamber depth (ACD) was deeper, the lens thickness (LT) was thinner, the mean keratometric value was lower, the WTW corneal diameter was longer, and the total corneal irregular astigmatism (TCIA) was lower in the long AXL group followed by the normal AXL and short AXL groups (*P* < 0.001). The keratometric astigmatism was highest in the long AXL group followed by the short AXL and normal AXL groups (*P* < 0.001).

**Table 1:** Preoperative and postoperative data of enrolled patients.

	All (n = 2018)	Short AXL (n = 260)	Normal AXL (n = 1660)	Long AXL (n = 98)	*P* value
Age (years)	58.60 ± 5.61	59.13 ± 5.55	58.69 ± 5.56	55.69 ± 5.93	< 0.001
Sex, male (%)	1006 (49.7)	142 (54.6)	811 (48.6)	53 (54.1)	0.867
AXL (mm)	23.76 ± 1.19	22.11 ± 0.34	23.84 ± 0.84	26.75 ± 0.73	< 0.001
ACD (mm)	3.17 ± 0.56	2.78 ± 0.28	3.21 ± 0.58	3.52 ± 0.28	< 0.001
LT (mm)	4.44 ± 0.32	4.61 ± 0.31	4.42 ± 0.31	4.34 ± 0.32	< 0.001
Km (mm)	44.17 ± 1.35	45.35 ± 1.15	44.03 ± 1.29	43.54 ± 1.23	< 0.001
Ka (D)	− 0.82 ± 0.54	− 0.92 ± 0.65	− 0.79 ± 0.50	− 1.09 ± 0.80	< 0.001
WTW (mm)	11.40 ± 0.38	11.15 ± 0.40	11.43 ± 0.37	11.58 ± 0.35	< 0.001
TCIA (μm)	0.16 ± 0.07	0.17 ± 0.07	0.15 ± 0.07	0.14 ± 0.08	< 0.001
**Preoperative visual acuity**					
UDVA, LogMAR	0.34 ± 0.30	0.42 ± 0.30	0.32 ± 0.30	0.28 ± 0.11	< 0.001
CDVA, LogMAR	0.03 ± 0.10	0.02 ± 0.06	0.03 ± 0.10	0.05 ± 0.13	0.008
UNVA, LogMAR	0.46 ± 0.19	0.44 ± 0.19	0.47 ± 0.19	0.46 ± 0.22	0.113
CNVA, LogMAR	0.01 ± 0.05	0.00 ± 0.02	0.01 ± 0.05	0.01 ± 0.04	0.116
Preoperative SE (D)	− 0.54 ± 2.66	2.00 ± 1.17	− 0.04 ± 2.17	− 6.41 ± 3.24	< 0.001
**Postoperative 6 m visual acuity**					
UDVA, LogMAR	0.02 ± 0.06	0.02 ± 0.07	0.02 ± 0.05	0.02 ± 0.07	0.410
CDVA, LogMAR	0.00 ± 0.04	0.00 ± 0.02	0.00 ± 0.04	0.00 ± 0.00	0.633
UNVA, LogMAR	0.02 ± 0.05	0.02 ± 0.04	0.02 ± 0.05	0.01 ± 0.03	0.176
CNVA, LogMAR	0.00 ± 0.02	0.00 ± 0.02	0.00 ± 0.02	0.00 ± 0.01	0.708
Postoperative 6 m SE (D)	− 0.10 ± 0.38	− 0.04 ± 0.39	− 0.10 ± 0.38	− 0.23 ± 0.32	< 0.001

*ACD* anterior chamber depth, *AXL* axial length, *CDVA* corrected distance visual acuity, *CNVA* corrected near visual acuity, *D* diopter, *IOL* intraocular lens, *Ka* keratometic astigmatism, *Km* mean keratometric value, *LT* lens thickness, *SE* spherical equivalent, *TCIA* total corneal irregular astigmatism, *UDVA* uncorrected distance visual acuity, *UNVA* uncorrected near visual acuity, *WTW* white to white corneal diameter.

ANOVA was used to evaluate whether there is a significant difference between three groups. Bonferroni HSD test wast used for post hoc analysis.

**Figure 1:**
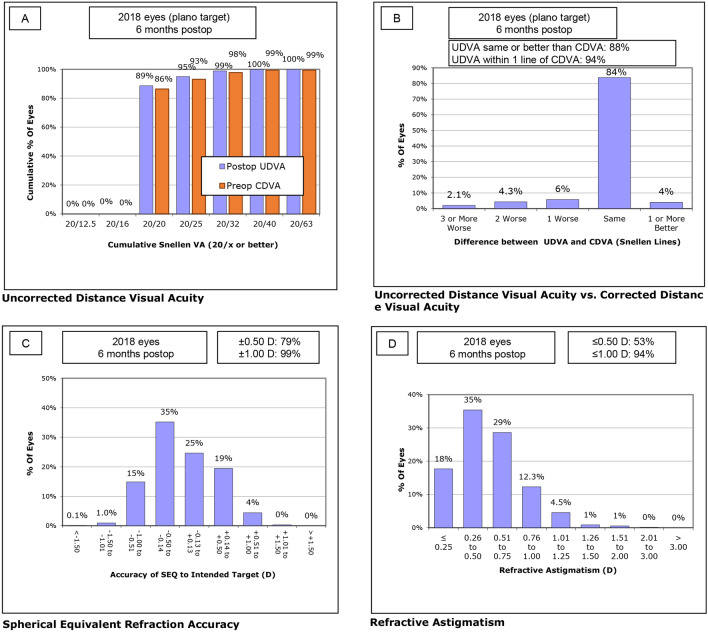
Refractive outcome at postoperative month 6 for the entire study population. (**A**) Uncorrected distance visual acuity, (**B**) uncorrected distance visual acuity versus corrected distance visual acuity, (**C**) spherical equivalent refraction accuracy, (**D**) refractive astigmatism. *CDVA* corrected distance visual acuity, *D* diopter, *UDVA* uncorrected distance visual acuity.

**Figure 2:**
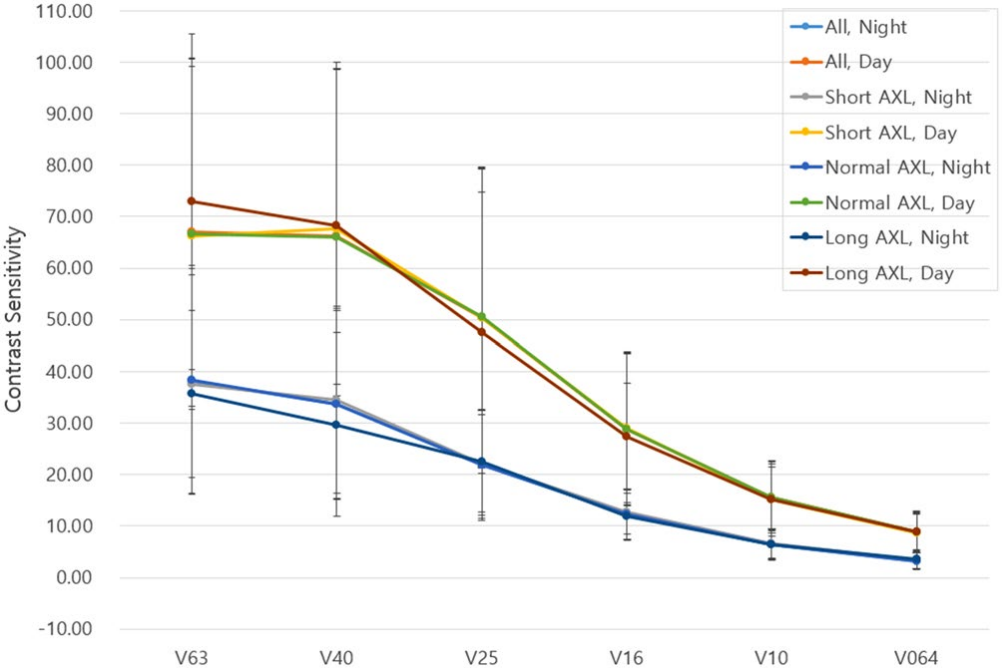
Bar graphs showing the contrast sensitivity under photopic and mesopic conditions at postoperative month 6. *Represents *P* value < 0.05.

At month 6, UDVA and UNVA were improved to 0.02 ± 0.06 and 0.02 ± 0.05, respectively, with a mean SE of − 0.10 ± 0.38 D. Figure 1 describes the refractive changes at month 6 in overall population. Although no difference was detected in the UDVA, CDVA, UNVA, and CNVA between groups (*P* = 0.410, *P* = 0.633, *P* = 0.176, and *P* = 0.708, respectively), the mean SE showed a statistically significant difference between groups. The short AXL group had the most hyperopic SE (− 0.04 ± 0.39 D), whereas the long AXL group had the most myopic SE (− 0.23 ± 0.32D; *P* < 0.001, see Table 1). There was no significant difference in the HOA parameters (Table 2) or the contrast sensitivity in every visual angle under scotopic and photopic conditions between groups (Fig. 2).

**Table 2:** High order aberration parameters after surgery.

Parameter	Short AXL (n = 260)	Normal AXL (n = 1660)	Long AXL (n = 98)	*P* value
Total	0.66 ± 0.24	0.71 ± 0.29	0.70 ± 0.24	0.405
Tilt (S1)	0.33 ± 0.21	0.32 ± 0.21	0.30 ± 0.16	0.816
High	0.35 ± 0.15	0.38 ± 0.22	0.38 ± 0.18	0.742
T. Coma	0.14 ± 0.07	0.14 ± 0.08	0.13 ± 0.06	0.631
T. Trefoil	0.24 ± 0.13	0.26 ± 0.16	0.27 ± 0.15	0.610
T. Sph	0.06 ± 0.05	0.07 ± 0.06	0.06 ± 0.03	0.359
Strehl ratio	0.05 ± 0.02	0.04 ± 0.03	0.04 ± 0.03	0.203
Area ratio 5 mm	50.16 ± 11.77	47.12 ± 12.61	45.43 ± 12.16	0.184
Area ratio 4 mm	57.29 ± 14.65	54.72 ± 15.20	53.72 ± 15.12	0.464

*MTF* modulation transfer function, *PSF* point spread function, *RMS HOA* root mean square of high-order aberrations, *SA* spherical aberration.

Refractive outcomes were compared in Table 3 (without adjusting mean RPE to zero) and Table 4 (after adjusting mean RPE to zero). The study MAE was lowest when using the Barrett Universal II formula with and without adjustment (0.33 ± 0.26 without adjustment; 0.30 ± 0.24 with adjustment). However, the MAE using the Barrett Universal II formula showed a significant difference between each group (*P* < 0.001), and the short AXL group showed a statistically higher MAE than the normal AXL or long AXL groups both with and without adjustment (*P* < 0.001). In subgroup analyses, the Barrett Universal II formula showed the lowest MAE in the normal AXL group (0.32 ± 0.25 without adjustment; 0.30 ± 0.23 with adjustment), whereas the Hoffer Q formula showed the lowest MAE in the short AXL group (0.34 ± 0.25 without adjustment; 0.32 ± 0.21 with adjustment). The Haigis formula showed the lowest MAE in the long AXL group (0.22 ± 0.18 without adjustment; 0.24 ± 0.19 with adjustment). The MAE was lowest in the long AXL group and highest in the short AXL group when using almost every IOL formula, which suggests greater difficulty in meeting refractive targets in eyes with short or long AXL.

**Table 3:** Refractive outcomes according to IOL formulars without adjusting mean RPE to zero.

	All population (n = 2018)	Short AXL (n = 260)	Normal AXL (n = 1660)	Long AXL (n = 98)	*P* value
**Barrett Universal II**					
Mean RPE ± SD	0.13 ± 0.40	0.36 ± 0.39	0.11 ± 0.01	− 0.09 ± 0.40	< 0.001
Range	(− 1.35, + 1.68)	(− 0.45, + 1.33)	(− 1.35, + 1.68)	(− 0.67, + 0.62)	
Post hoc analysis		S vs N; *P* < 0.001, N vs L; *P* < 0.001, S vs L; *P* < 0.001			
MAE ± SD	0.33 ± 0.26	0.44 ± 0.30	0.32 ± 0.25	0.26 ± 0.18	< 0.001
Post hoc analysis		S vs N; *P* < 0.001, N vs L; *P* = 0.049, S vs L; *P* < 0.001			
MedAE	0.28	0.40	0.27	0.21	
Within ± 0.50 D, eye (%)	1567 (79.31)	157 (61.54)	1327 (81.41)	83 (87.37)	
**SRK T**					
Mean RPE ± SD	0.04 ± 0.45	0.18 ± 0.42	0.02 ± 0.45	− 0.03 ± 0.37	< 0.001
Range	(− 1.58, + 1.64)	(− 1.08, + 1.64)	(− 1.58, + 1.57)	(− 0.92, + 0.94)	
Post hoc analysis		S vs N; *P* < 0.001, N vs L; *P* = 0.520, S vs L; *P* < 0.001			
MAE ± SD	0.35 ± 0.28	0.36 ± 0.27	0.36 ± 0.28	0.29 ± 0.22	0.087
Post hoc analysis		S vs N; *P* = 0.795, N vs L; *P* = 0.075, S vs L; *P* = 0.059			
MedAE	0.31	0.32	0.31	0.24	
Within ± 0.50 D, eye (%)	1588 (79.79)	202 (78.29)	1304 (79.51)	82 (89.13)	
**Holladay 1**					
Mean RPE ± SD	0.17 ± 0.40	0.27 ± 0.38	0.14 ± 0.40	0.27 ± 0.33	< 0.001
Range	(− 1.44, + 1.65)	(+ 1.38, − 0.57)	(− 1.44, + 1.65)	(− 0.54, + 0.92)	
Post hoc analysis		S vs N; *P* < 0.001, N vs L; *P* = 0.011, S vs L; *P* = 0.979			
MAE ± SD	0.34 ± 0.27	0.38 ± 0.26	0.34 ± 0.27	0.35 ± 0.25	0.084
Post hoc analysis		S vs N; *P* = 0.032, N vs L; *P* = 0.889, S vs L; *P* = 0.655			
MedAE	0.30	0.34	0.37	0.30	
Within ± 0.50 D, eye (%)	1283 (77.61)	171 (73.71)	1062 (78.49)	50 (73.53)	
**Hoffer Q**					
Mean RPE ± SD	0.18 ± 0.38	0.16 ± 0.39	0.17 ± 0.39	0.25 ± 0.31	0.209
Range	(− 1.50, + 1.57)	(− 0.73, + 0.99)	(− 1.50, + 1.57)	(− 0.45, + 1.04)	
Post hoc analysis		S vs N; *P* = 0.958, N vs L; *P* = 0.199, S vs L; *P* = 0.220			
MAE ± SD	0.34 ± 0.25	0.34 ± 0.25	0.34 ± 0.25	0.30 ± 0.26	0.368
Post hoc analysis		S vs N; *P* = 0.987, N vs L; *P* = 0.347, S vs L; *P* = 0.393			
MedAE	0.29	0.30	0.29	0.22	
Within ± 0.50 D, eye (%)	1346 (80.64)	164 (79.61)	1121 (80.88)	61 (79.22)	
**Haigis**					
Mean RPE ± SD	0.16 ± 0.38	0.27 ± 0.43	0.16 ± 0.37	0.02 ± 0.28	0.004
Range	(− 1.245, + 1.17)	(− 0.74, + 1.11)	(− 1.25, + 1.08)	(− 0.39, + 0.85)	
Post hoc analysis		S vs N; *P* = 0.030, N vs L; *P* = 0.115, S vs L; *P* = 0.004			
MAE ± SD	0.34 ± 0.27	0.42 ± 0.29	0.33 ± 0.24	0.22 ± 0.18	< 0.001
Post hoc analysis		S vs N; *P* = 0.008, N vs L; *P* = 0.032, S vs L; *P* = < 0.001			
MedAE	0.28	0.31	0.28	0.15	
Within ± 0.50 D, eye (%)	555 (81.73)	53 (67.95)	471 (82.92)	31 (93.94)	

ANOVA was used to evaluate whether there is a significant difference between three groups. Bonferroni HSD test wast used for post hoc analysis.

*CDVA* corrected distance visual acuity, *CNVA* corrected near visual acuity, *S* short AXL, *L* long AXL, *MAE* mean absolute error, *MedAE* median absolute error, *N* normal AXL, *RPE* refractive prediction error, *SE* spherical equivalent, *UDVA* uncorrected distance visual acuity, *UNVA* uncorrected near visual acuity.

**Table 4:** Refractive outcomes according to IOL formulars after adjusting mean RPE to zero.

	All (n = 2018)	Short AXL (n = 260)	Normal AXL (n = 1660)	Long AXL (n = 98)	*P* value
**Barrett Universal II**					
Mean RPE ± SD	0.00 ± 0.39	0.23 ± 0.39	− 0.02 ± 0.38	− 0.22 ± 0.30	< 0.001
Range	(− 1.48, + 1.55)	(− 0.58, + 1.20)	(− 1.48, + 1.55)	(− 0.80, + 0.49)	
Post hoc analysis		*P* < 0.001 for all analysis			
MAE ± SD	0.30 ± 0.24	0.37 ± 0.26	0.30 ± 0.23	0.30 ± 0.22	< 0.001
Post hoc analysis		S vs N; *P* < 0.001, N vs L; *P* = 0.998, S vs L; *P* = 0.067			
MedAE	0.26	0.33	0.25	0.27	
Within ± 0.50 D, eye (%)	1575 (79.46)	179 (70.75)	1322 (80.81)	74 (79.57)	
**SRK T**					
Mean RPE ± SD	0.00 ± 0.43	0.14 ± 0.42	0.02 ± 0.44	− 0.06 ± 0.36	< 0.001
Range	(− 1.62, + 1.60)	(− 1.12, + 1.60)	(− 1.62, + 1.58)	(− 0.96, + 0.90)	
Post hoc analysis		S vs N; *P* < 0.001, N vs L; *P* = 0.616, S vs L; *P* < 0.001			
MAE ± SD	0.35 ± 0.26	0.35 ± 0.27	0.35 ± 0.26	0.29 ± 0.22	0.139
Post hoc analysis		S vs N; *P* = 0.993, N vs L; *P* = 0.171, S vs L; *P* = 0.121			
MedAE	0.30	0.31	0.30	0.26	
Within ± 0.50 D, eye (%)	1473 (75.46)	189 (74.70)	1208 (75.17)	76 (82.61)	
**Holladay 1**					
Mean RPE ± SD	0.00 ± 0.39	0.10 ± 0.38	− 0.03 ± 0.39	0.11 ± 0.33	< 0.001
Range	(− 1.61, + 1.48)	(− 0.74, + 1.21)	(− 1.61, + 1.48)	(− 0.71, + 0.75)	
Post hoc analysis		S vs N; *P* < 0.001, N vs L; *P* = 0.011, S vs L; *P* = 0.979			
MAE ± SD	0.31 ± 0.24	0.32 ± 0.22	0.31 ± 0 24	0.28 ± 0.20	0.507
Post hoc analysis		S vs N; *P* = 0.965, N vs L; *P* = 0.507, S vs L; *P* = 0.493			
MedAE	0.26	0.27	0.26	0.23	
Within ± 0.50 D, eye (%)	1320 (80.39)	188 (80.69)	1075 (80.16)	57 (83.82)	
**Hoffer Q**					
Mean RPE ± SD	0.00 ± 0.38	− 0.02 ± 0.39	− 0.01 ± 0.38	0.07 ± 0.31	0.223
Range	(− 1.68, + 1.39)	(− 0.91, + 0.81)	(− 1.68, + 1.39)	(− 0.63. + 0.86)	
Post hoc analysis		S vs N; *P* = 0.936, N vs L; *P* = 0.220, S vs L; *P* = 0.225			
MAE ± SD	0.30 ± 0.23	0.32 ± 0.21	0.30 ± 0.23	0.26 ± 0.19	0.124
Post hoc analysis		S vs N; *P* = 0.568, N vs L; *P* = 0.210, S vs L; *P* = 0.102			
MedAE	0.26	0.28	0.26	0.20	
Within ± 0.50 D, eye (%)	1338 (81.34)	159 (77.56)	1116 (81.70)	63 (85.14)	
**Haigis**					
Mean RPE ± SD	0.00 ± 0.38	0.11 ± 0.43	− 0.01 ± 0.37	− 0.13 ± 0.28	0.004
Range	(− 1.41, + 1.01)	(− 0.90, + 0.95)	(− 1.41, + 1.01)	(− 0.55, + 0.69)	
Post hoc analysis		S vs N; *P* = 0.029, N vs L; *P* = 0.138, S vs L; *P* = 0.005			
MAE ± SD	0.31 ± 0.23	0.36 ± 0.25	0.31 ± 0.23	0.24 ± 0.19	0.019
Post hoc analysis		S vs N; *P* = 0.036, N vs L; *P* = 0.495, S vs L; *P* = 0.041			
MedAE	0.27	0.29	0.27	0.25	
Within ± 0.50 D, eye (%)	550 (82.58)	53 (70.67)	469 (83.90)	28 (87.50)	

ANOVA was used to evaluate whether there is a significant difference between three groups. Bonferroni HSD test wast used for post hoc analysis.

*CDVA* corrected distance visual acuity, *CNVA* corrected near visual acuity, *S* short AXL, *L* long AXL, *MAE* mean absolute error, *MedAE* median absolute error, *N* normal AXL, *RPE* refractive prediction error, *SE* spherical equivalent, *UDVA* uncorrected distance visual acuity, *UNVA* uncorrected near visual acuity.

**Figure 3:**
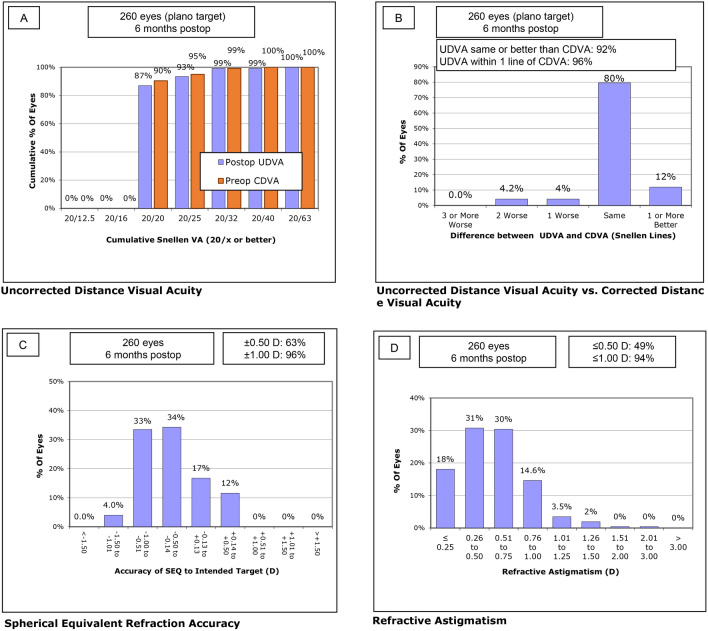
Refractive outcome at postoperative month 6 for the short AXL group. (**A**) Uncorrected distance visual acuity, (**B**) uncorrected distance visual acuity versus corrected distance visual acuity, (**C**) spherical equivalent refraction accuracy, (**D**) refractive astigmatism. *CDVA *corrected distance visual acuity, *D* diopter, *UDVA* uncorrected distance visual acuity.

**Figure 4:**
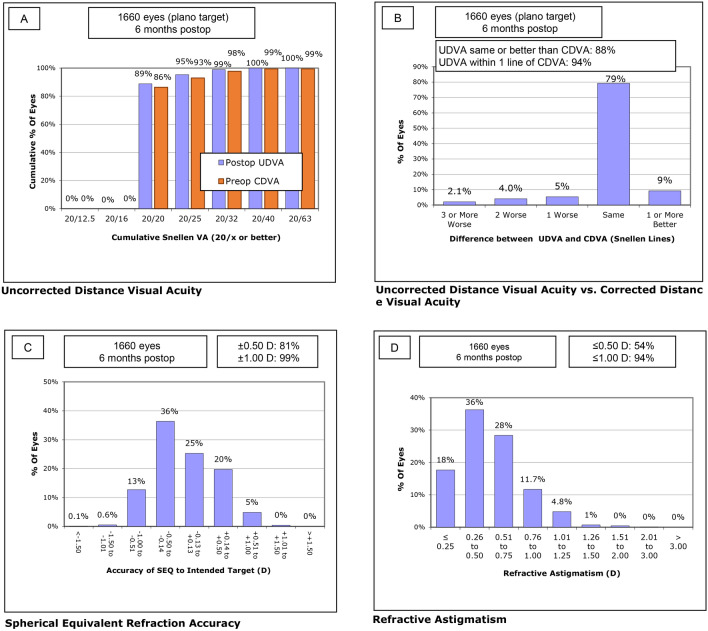
Refractive outcome at postoperative month 6 for the normal AXL group. (**A**) Uncorrected distance visual acuity, (**B**) uncorrected distance visual acuity versus corrected distance visual acuity, (**C**) spherical equivalent refraction accuracy, (**D**) refractive astigmatism. *CDVA* corrected distance visual acuity, *D* diopter, *UDVA* uncorrected distance visual acuity.

**Figure 5:**
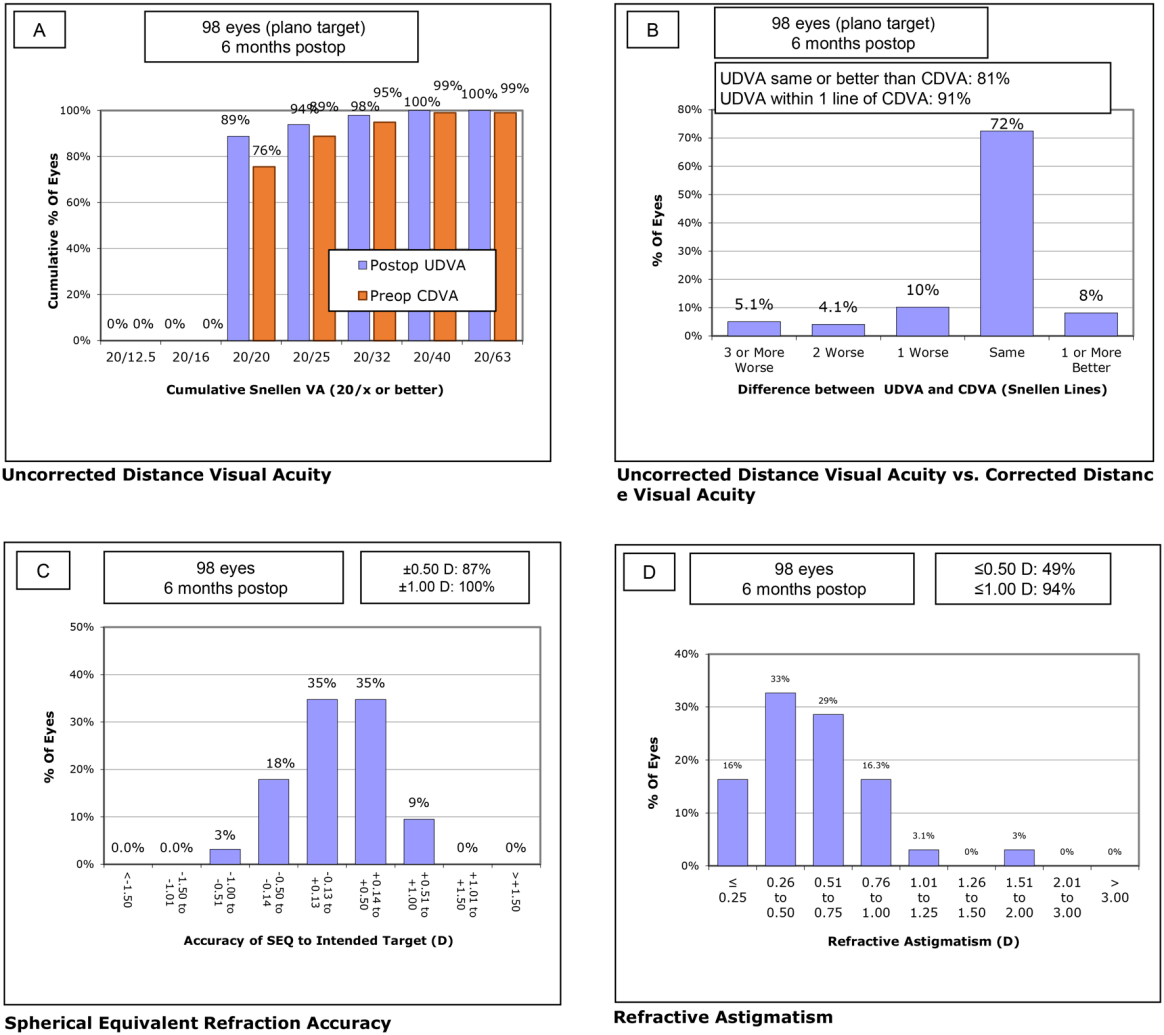
Refractive outcome at postoperative month 6 for the long AXL group. (**A**) Uncorrected distance visual acuity, (**B**) uncorrected distance visual acuity versus corrected distance visual acuity, (**C**) spherical equivalent refraction accuracy, (**D**) refractive astigmatism. *CDVA* corrected distance visual acuity, *D* diopter, *UDVA* uncorrected distance visual acuity.

**Figure 6:**
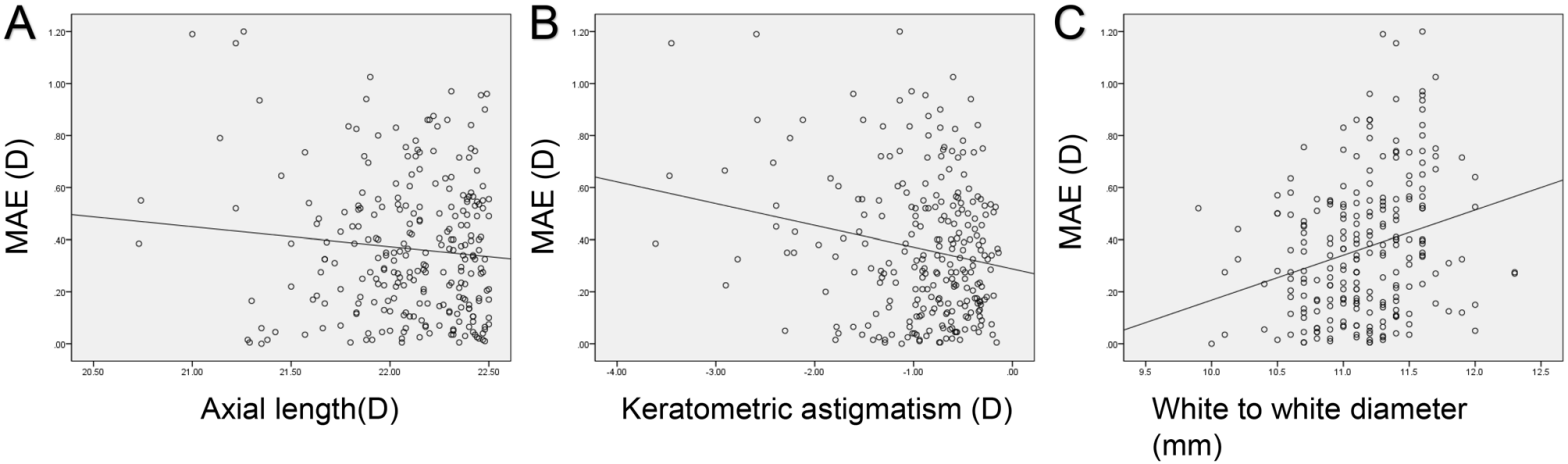
Dot graph for the association between mean absolute error (MAE) from the Barrett Universal II formula and keratometric astigmatism (**A**), white-to-white (WTW) corneal diameter (**B**), and axial length (AXL; **C**).

Figures 3, 4 and 5 shows the refractive changes at month 6 for each group. The short AXL group showed the highest proportion of positive RPE: 22.0% of the eyes showed an RPE higher than + 0.50 D, when compared 18.0% from the normal AXL group and 21.0% from the long AXL group. To evaluate the factors that were associated with a high postoperative RPE in the short AXL group, univariate and multivariate analyses were done (Table 5). The AXL and Ka were independently associated with a high RPE using the Barrett Universal II formula in both data with and without adjustment (*P* = 0.001 and *P* = 0.001, respectively, for data without adjustment and *P* = 0.016 and *P* = 0.013, respectively, for data with adjustment, Fig. 6A,B). In the data with adjustment, the WTW corneal diameter also showed an independent association with a high RPE using the Barrett Universal II formula (Fig. 6C), in addition to the AXL and Ka.

**Table 5:** Univariate and multivariate analysis for high MAE without and with adjusting mean RPE to zero in short AXL group (n = 260).

	Without adjustment				With adjustment			
	Univariate		Multivariate R^2^ = 0.012		Univariate		Multivariate R^2^ = 0.122	
	R	*P*	β	*P*	R	*P*	β	*P*
Age (years)	0.020	0.372			− 0.036	0.277		
Sex, male	0.050	0.167			− 0.048	0.280		
SE (D)	0.026	0.242			0.059	0.349		
AXL (mm)	− 0.073	0.001	− 0.082	0.001	− 0.097	0.120	− 0.157	0.016
ACD (mm)	− 0.056	0.011			0.003	0.957		
LT (mm)	0.050	0.025			0.070	0.268		
Km (mm)	0.061	0.006			0.008	0.895		
Ka (D)	− 0.059	0.009	− 0.081	0.001	− 0.196	0.002	− 0.163	0.013
WTW (mm)	− 0.046	0.049			0.262	< 0.001	0.254	< 0.001
TCIA (μm)	0.028	0.216			− 0.085	0.174		
Pupil diamter (mm)	− 0.007	0.752			− 0.057	0.371		

*ACD* anterior chamber depth, *AXL* axial length, *D* diopter, *IOL* intraocular lens, *Ka* keratometric astigmatism, *Km* mean keratometry, *LT* lens thickness, *SE* spherical equivalent, *TCIA* total corneal irregular astigmatism, *WTW* white to white corneal diameter.

## Discussion

In the present study, we evaluated the accuracy of IOL formulas for Panoptix implantation in a relatively large cohort. Our data suggest fair refractive outcomes: 2003 (99.3%) of the 2018 study eyes had an SE result within ± 1.00 D of the predicted outcome. As mentioned earlier, we use different IOL formulas for TFNT implantation according to the AXL based on our experiences. No single IOL formula showed RPE results comparable to our actual SE results, suggesting a variation in IOL formula accuracy based on the AXL for the TFNT IOL.

When we compared built-in IOL formulas in the IOLMaster 700, namely, the SRK/T, Hoffer Q, Holladay 1, Haigis, and Barrett Universal II, we found that the overall accuracy for the entire study population was best when using the Barrett Universal II formula, which is consistent with previous studies^2,6,8,11^. However, the Barrett Universal II formula, as well as other IOL formulas, yielded myopic predictions. This is consistent with a previous study by Gökce et al. that reported that the percentage of eyes within + 0.50 D of the RPE was around 70%^9^. The accuracy of the IOL formula in hyperopic eyes has been controversial, as the calculation of the effective lens position (ELP) in a short eye is important and difficult at the same time, due to the short distance between IOL and fovea^12^. Many studies about the accuracy of IOL formulas for short eyes emphasized that the Hoffer Q^13^, Holladay 1^3,13^, or Barrett Universal II are the best to use in such eyes^2^. In the present study, which focused only on the TFNT IOL and IOLMaster 700, the Holladay 1 and Hoffer Q formulas showed better performance in short eyes than the Barrett Universal II formula. According to our subanalysis, higher astigmatism, wider WTW corneal diameter, and shorter AXL were associated with poor performance of the Barrett Universal II formula in short eyes.

It is of particular interest that the third generation Hoffer Q and Holladay 1 IOL formulas, which use two input variables (AXL and keratometry), performed better than the fifth generation IOL formula. The Barrett Universal II formula uses three input variables that represent the anterior segment characteristics (ACD, LT, and WTW corneal diameter) in addition to the AXL and keratometry. These anterior segment variables are not proportional to the AXL in small eyes when compared with eyes with normal of long AXL^14^. This high variability in the proportion of ocular parameters in small eyes may result in an inaccurate calculation of ELP. We speculate that short eyes with atypical proportion, such as wide WTW corneal diameter for its short AXL, may run the higher risk of an inaccurate prediction using the Barrett Universal II formula than the short eyes with typical proportion.

Every IOL formula evaluated in the present study showed good performance in myopic eyes in contrast with hyperopic eyes, which suggests a greater ease in IOL target prediction in myopic eyes. Among the IOL formulas evaluated in the present study, the Haigis formula performed the best for myopic eyes, which is consistent with previous studies^15–17^.

A limitation of the present study is that the surgery was performed by two surgeons, and the refraction was performed by multiple practitioners. Because the data were focused on a single IOL type in the Asian population, study results may not be generalized to other types of IOL models or different ethnic groups. In addition, because the range of available TFNT IOLs are limited to between 6.0 and 34.0 D, this dataset does not include extreme myopia. So far, to our knowledge, this is the largest retrospective study of refractive outcomes of the TFNT IOL implant. Our findings suggest that the Holladay 1 and Hoffer Q formulas would provide the best refractive outcome in hyperopic eyes, and the Haigis formula would provide the best refractive outcome in myopic eyes. Overall outcomes were best when using the Barrett Universal II formula.

## Methods

We performed a retrograde chart review of eyes that underwent phacoemulsification and lens implantation with the TFNT IOL during the period of January 3rd, 2019, to February, 28th, 2020, at Keye Eye Center, Seoul, Korea. All eye surgeries were performed by two experienced surgeons (H.K. and S.J.) using a standard sutureless 2.2-mm microincision. A 5.2-mm diameter continuous curvilinear capsulorrhexis was performed with femtosecond laser (LENSAR, Orlando, Florida, United States) before corneal incision. Eyes with poor retinal function due to conditions such as age-related macular degeneration, diabetic retinopathy, or retinal vascular occlusions were not subjected to MIOL implantation. Eyes with preoperative corrected distance visual acuity (CDVA) less than 20/40, previous corneal or vitreoretinal surgery, intraoperative capsular damage, or any kind of postoperative complication, such as cystoid macular edema (CME), were excluded from the analysis. The Institutional Review Board (IRB)/Ethics Committee of KEYE EYE Center approved the study (IRB number P12361001-001) and waived the requirement for informed consent because of the retrospective nature of the study. The study protocol adhered to the tenets of the Declaration of Helsinki.

### IOL power calculation and outcome measure

In most of the study cases, the IOL power was selected to target emmetropia by choosing the first negative power IOL using the Barrett Universal II formula in the IOLMaster 700 (Carl Zeiss Meditec). However, we tend to choose more negatively targeted IOLs in hyperopic eyes and more positively targeted IOLs in myopic eyes. Eyes with corneal astigmatism higher than 0.50 D were recommended for toric IOL implantation at our institution.

The primary goal of this study was to evaluate the accuracy of IOL formulas based on the AXL. Eyes were subcategorized as hyperopic (AXL ≤ 22.5 mm), normal (22.5 mm < AXL ≤ 26.0 mm), and myopic (AXL > 26.00 mm). The accuracy of theoretical IOL formulas for each group was analyzed as previously recommended^18^. Candidate formulas used were the Barrett Universal II, Sanders-Retzlaff-Kraff/Theoretical (SRK/T), Holladay 1, Hoffer Q, and Haigis, all of which came preinstalled on the IOLMaster 700. Lens constant optimizations for the TFNT IOL were performed in collaboration with Carl Zeiss Meditec AG.

Postoperative visual and refractive outcomes were described as previously reported (https://www.londonvisionclinic.com/refractivesurgeryoutcomes/)19. The refractive prediction error (RPE) was calculated by subtracting the predicted refractive error from the SE at postoperative month 6. A negative RPE indicates a more myopic result than the predicted refractive error. To eliminate the systemic myopic or hyperopic prediction error, the mean RPE from each IOL formula was zeroed out by adjusting the RPE for each eye up or down by an amount equal to the mean RPE in that group. Because there is controversy about the optimization to be used in atypical eyes, we evaluated the MAE and median absolute error (MedAE) both with and without the adjustment. The MAE was calculated by averaging the absolute differences between the SE at 6 months and the predicted refractive error. The MedAE was chosen as the central value of the absolute errors. The number and percentage of eyes within ± 0.25 D, ± 0.50 D, ± 0.75 D, and ± 1.00 D of RPE were evaluated.

Monocular uncorrected distance visual acuity (UDVA), CDVA, uncorrected near visual acuity (UNVA) and corrected near visual acuity (CNVA) were checked at postoperative months 1, 2, and 6 using the decimal system and converted into LogMAR for statistical analysis. Near visual acuities were measured using the Sloan ETDRS Format Near Vision chart 3 with 100% contrast under photopic conditions (85 candelas [cd]/m^2^) at 40 cm. The intraocular optical quality of the IOL was estimated by calculating the higher order aberrations (HOAs) using OPD scan III and OPD Station software (NIDEK Co. Ltd., Aichi, Japan). Ocular, corneal, and internal root mean square (RMS) of the HOAs and ocular, corneal, and internal Zernike coefficients of second-, third-, and fourth-order aberrations were calculated. The Strehl ratio of the point spread function (PSF) and the modulation transfer function (MTF) from the postoperative RMS of the total ocular wave aberration Z (1 ≤ n ≤ 8) were assessed at a pupil diameter of 5.0 mm. Contrast sensitivities were evaluated at postoperative month 6 using the CGT-2000 instrument (Takagi. Seiko co., Ltd., Nagano-Ken, Japan) under an illumination of 85 cd/m^2^.

### Statistical analysis

Statistical analyses were conducted using SPSS, version 15.0 for Windows (SPSS, Inc., Chicago, IL, United States). Descriptive data were recorded as mean ± SD unless otherwise specified. The Shapiro–Wilk test was used to assess normality of the continuous variables. An analysis of variance (ANOVA) was used for the comparison of three or more data. Bonferroni test was used for post hoc analysis. The Pearson correlation coefficient was determined to assess the association between continuous variables, according to the normality of distribution. Independent variables significantly associated with scores in univariate analyses (*P* < 0.05) and potentially confounding parameters were included as independent covariables in multivariate analyses by multiple regression analysis. All *P* values were 2-sided, and a *P* value < 0.05 was considered statistically significant.
